# Chancre of the Eyelid and Caruncle

**Published:** 1885-04

**Authors:** E. L. Holmes

**Affiliations:** Professor of Diseases of the Eye and Ear, Rush Medical College; 207 Clark street


ARTICLE IV.

### CASES OF CHANCRE OF THE EYELID AND OF THE CARUNCLE.

*Reported to the* CHICAGO OPHTHALMOLOGICAL AND OTOLOGICAL SOCIETY. *By* E. L. HOLMES, A. M., M. D., *Professor of Diseases of the Eye and Ear, Rush Medical College.*

I have observed three cases in which the initial lesion of syphilis was located on the lids, and one in which it was on the caruncle. The infrequency of the location, and the occasional difficulty in the diagnosis, add to the interest of such complications. I have retained the history of but two of the cases, in which the lid was involved.

In 1865, Mr. M., 63 years of age, came to me with a characteristic ulcer of the left upper lid, which had slowly increased in size for a month, until it had destroyed a large triangular portion of the free border of the lid through the cartilage and integument.

The indurated and elevated edges were characteristic, and rendered a diagnosis almost certain at the first glance.

The pre-auricular glands were slightly enlarged and tender. The patient would not admit any known exposure. By the use of calomel locally, and *pilula hydrargyri* internally, the ulcer healed speedily, leaving a large notch in the lid. Although the patient was urged to continue the remedies many months, he returned at the end of three months with characteristic appearances of the throat and integument.

The second case presented complications which obscured the diagnosis. A young man, about 30 years of age, while employed in rolling rails, received a "flash," which burned his left side and arm, and also his upper lid, a drop of hot slag having been received in the fold of the integument, and held in place by a spasm of the orbicularis and muscles of the face.

A few days after the accident, the lid was found to be red, and much swollen, presenting a dark-colored round spot, about a quarter of an inch in diameter, resembling a deep burn, (moxa).

I informed the patient that he would probably soon recover without much, if any, deformity of the lid, and simply prescribed vaseline dressings. In a few days the eschar was thrown off, leaving quite a deep ulceration on the swollen lid. I saw the patient at the end of two weeks, and was surprised to observe how little tendency there was for the ulcer to heal. At the end of two weeks more, there was no special change. There was the same amount of swelling and ulceration. My attention, however, was not attracted to anything characteristic in the lesion.

After three weeks, the general swelling of the lid began to recede, while the ulcer showed hard elevated edges—symptoms which might arouse the strongest suspicions. My colleagues at the Eye and Ear Infirmary, to whom I submitted the case for examination, concurred in the opinion that the ulcer was true chancre. The patient presented no evidence of infection, either on the genitals or other portions of the integument, and was unable to explain the complication. The pre-auricular and cervical glands were overlooked.

I must confess I am unable to form an opinion regarding the time when the burn became infected, or at what time a more experienced observer would have detected the dangerous nature of the affection.

It was decided, inasmuch as the ulcer was not encroaching on the free border of the lid, to continue palliative treatment and await developments. After ten days, characteristic roseola appeared, when the calomel was prescribed locally for the ulcer and *pilula hydrargyri* internally. The ulcer soon healed, with scarcely any deformity of the lid.

In the summer of 1882, a young physician came casually under my observation, while he was under the treatment of Dr. Montgomery. Subsequently he came temporarily under my care during the absence of Dr. M. For about four weeks there was simply an inflammation of the ocular conjunctiva at the internal angle, especially implicating the caruncle. This was very red and hypertrophied, with no appearance at any time of ulceration. The caruncle was in the condition of enlargement not unfrequently seen in certain cases of acute conjunctivitis. After three weeks, the pre-auricular and cervical glands became noticeably tender and enlarged. I had not the slightest suspicion of local infection, but, without questioning the patient, entertained the possibility that the local trouble was rebellious to treatment in consequence of constitutional infecfection, and urged the use of mercurials. The patient took *hydrargyri bichloridum* for a short period with benefit, but abandoned it. Professor Hyde, whose opinion was now sought, pronounced an unqualified opinion that the swelling of the conjunctiva was the primary lesion of syphilis, and verified his diagnosis by discovering characteristic eruption on the body. Simple treatment relieved the local as well as the general symptoms.

Years ago I enjoyed the best of opportunities to study the initial lesion of syphilis. During the past eighteen years I have not, I think, observed a single case in addition to those here reported, and consequently for a time was entirely off my guard in the last two cases. These certainly presented difficulties of diagnosis, and could well deceive any one not accustomed to observe chancre in unusual localities with unusual aspects.

The difficulty would probably be reduced to the minimum if the practitioner would carefully examine the condition of the nearest lymphatic glands, and from time to time inspect the surface of the body.

The lymphatics in the vicinity of the chancre are nearly always tender and enlarged. The converse of this, however, is not true, for large benign abscesses of the upper lid, near the external angle, are very liable to affect the pre-auricular glands.

In view of possible danger to friends surrounding a patient with chancre of the eye, early diagnosis and treatment are matters of exceeding importance. Any obscure indurations on the eye or its appendages, with inflammation, accompanied by an affection of the glands near, should receive most careful investigation.

207 Clark street.

